# “I didn’t know it was going to be like this.”: unprepared for end-of-Life care, the experiences of care aides care in long-term care

**DOI:** 10.1186/s12904-023-01244-y

**Published:** 2023-09-09

**Authors:** Laura Booi, Judith Sixsmith, Habib Chaudhury, Deborah O’connor, Claire Surr, Melanie Young, Andrew Sixsmith

**Affiliations:** 1 Centre for Dementia Research, School of Health, Leeds Beckett University, Leeds Beckett University, CL521 Calverley Building, City Campus, Leeds, LS1 3HE UK; 2 School of Nursing and Health Sciences, University of Dundee, 11 Airlie Pl, Dundee, DD1 4HJ UK; 3 Department of Gerontology, Simon Fraser University, Suite #2800, Harbour Centre, 515 W Hastings Street, Vancouver, BC V6B 5K3 Canada; 4 School of Social Work, Centre for Research on Personhood in Dementia (CRPD), University of British Columbia, Jack Bell Building, 2080 West Mall, Co-Director, Vancouver, BC V6T 1Z2 Canada; 5 Kiwanis Village, Vancouver Island Health Authority, British Columbia, Canada; 6 Department of Gerontology, Simon Fraser University, Suite #2800, Harbour Centre, 515 W Hastings Street, Vancouver, British Columbia, V6B 5K3 Canada

**Keywords:** Care aides, Long-term care, Moral distress, End of life, Older adults, Palliative care, Dementia, Qualitative research, Work satisfaction, Working conditions

## Abstract

**Background:**

Care aides provide up to 70–90% of the direct care for residents in long-term care (LTC) and thus hold great potential in improving residents’ quality of life and end-of-life (EoL) care experiences. Although the scope and necessity of the care aide role is predicted to increase in the future, there is a lack of understanding around their perceptions and experiences of delivering EoL care in LTC settings. The aim of this study was to gain an understanding of the perspectives, experiences, and working conditions of care aides delivering end-of-life care in LTC in a rural setting, within a high-income country.

**Methods:**

Data were collected over ten months of fieldwork at one long-term care home in western Canada; semi-structured interviews (70 h) with 31 care aides; and observation (170 h). Data were analysed using Reflexive Thematic Analysis.

**Results:**

Two themes were identified: (i) the emotional toll that delivering this care takes on the care aids and; (ii) the need for healing and support among this workforce. Findings show that the vast majority of care aides reported feeling unprepared for the delivery of the complex care work required for good EoL care. Findings indicate that there are no adequate resources available for care aides’ to support the mental and emotional aspects of their role in the delivery of EoL care in LTC. Participants shared unique stories of their own self-care traditions to support their grief, processing and emotional healing.

**Conclusions:**

To facilitate the health and well-being of this essential workforce internationally, care aides need to have appropriate training and preparation for the complex care work required for good EoL care. It is essential that mechanisms in LTC become mandatory to support care aides’ mental health and emotional well-being in this role. Implications for practice highlight the need for greater care and attention played on the part of the educational settings during their selection and acceptance process to train care aides to ensure they have previous experience and societal awareness of what care in LTC settings entails, especially regarding EoL experiences.

## Introduction

Death in long-term care (LTC) facilities is inevitable, often described as the ‘last stop’ for residents since for the vast majority leave this setting only when they die [1]. There is a certainty is the growing need in LTC to deliver end-of-life (EoL) care. Care aides, known by many names including health care aides, personal support workers, care assistants, health assistants, nursing assistants, or nurses’ aides, are the backbone of the healthcare team in LTC, especially in relation to delivering EoL care [2].

In LTC, care aides provide upward of 70–90% of the direct care [3] and therefore, hold significant potential for impacting residents’ quality of life and EoL experience [4–6]. They are the largest unregulated workforce in health and social care globally in high-income countries, without any consistent educational standards, legally defined scope of practice, professional practice, professional conduct review process and regulatory or governing body [3, 7–10].

Care aides hold little autonomy in their role and are consistently reported as an understudied and underrepresented workforce in the international literature [2, 7–12]. As a workforce, care aides are 90% women, and in urban centres in high-income countries, they are prominently made up of migrant women [6, 10, 12–15]. For example, the Canadian workforce which is reflective of the international literature from high income countries [16], highlight care aides as predominantly women over the age of 40 who speak English as a second language [6, 14].

Internationally care aides face barriers that affect both their workplace satisfaction and the delivery of care. LTC care aides report experiencing daily verbal abuse and violence from residents [17, 18] and bullying and incivility from other members of the health care team [19]. They frequently report that their care is rushed and that, due to lack of time, tasks are omitted [20, 21]. In addition, consistently, the training and preparation care aides receive for their role in LTC is reported to be inadequate for the demands of their role [11, 22, 23]. With regard to EoL care training, in Canada care aides are taught such items such as ‘the dance’ educational program [11], where they are taught to not get too emotionally close to residents to protect themselves, but still deliver good quality care.

Internationally, death is a regular occurrence LTC, with projections showing LTC to become the most common place of death for older adults by 2040 [24]. As such, staff in LTC, including care aides, experience death, dying and grief as day-to-day experiences in their working life, yet research suggests they are often overlooked as requiring grief and bereavement support as death is considered part of their occupation [10, 25, 26]. Care aides report high rates of burnout and turnover [27], as well as moral distress [28]. Care aides experience grief at the loss of residents, yet the formal avenues for healing and support for care aides are minimal, with a lack of organizational support offered to help this workforce overcome grief, hence they depend on each other and themselves for this support [25, 29].

Thus, long before the global COVID-19 pandemic, warning alarms were raised regarding the lack of training and support surrounding care aides delivering EoL care in LTC and their high exposure to deaths [29, 30]. During the COVID-19 pandemic Canadian LTC facilities have the highest rates of excess deaths globally, and as such care aides had the highest exposure to deaths in these settings [31]. Although the data for this study was collected prior to the pandemic it shares valuable lessons on the experiences of care aides delivering EoL care that are essential for understanding the challenges this this workforce faces in providing EoL care and the negative implications they experience personally in this role. The aim of this study is to gain an understanding on the perspectives, experiences, and working conditions of care aides delivering EoL care in LTC in a rural setting, within a high-income country.

## Method

This is a qualitative study using both observations and interviews as data collection methods.

### Participant sampling and recruitment

The participant group for this study was care aides employed at a LTC facility in British Columbia, Canada. Convenience sampling was used for the first interviews and then maximum variation sampling to develop a diverse sample for number of years of care aide experience, gender, ethnicity, shiftwork and age [32]. Initially, participants were approached about the study with a study information sheet via the Director of Care and an in-person meeting with the researcher to discuss the aims of the study and the expectations as part of their participation.

Participants were aged between 26 and 55 years (average 42 years), twenty-six identified as female and five as male. Participants had two months to 32 years’ experience working in LTC (average = 9 years) and had worked in 2 to 8 different LTC settings (mean = 2.5 facilities). Twenty-six participants identified as White, two as Filipino, one as First Nation, one as Hispanic, and one as Chinese.

### Data collection

Semi-structured interviews with care aides employed at a LTC were conducted. Data were gathered by the first author over a ten-months and involved semi-structured interviews with a total of 31 care aides. Open ended questions about the care aide perspectives and experiences were used to facilitate conversation. Questions pertained to their role and experience of delivering care to residents. Each interview evolved into a different conversation, depending on the participant’s experience in LTC.

Participants received no personal benefit from participation; however, interviews were conducted during their work time with the consent of their employers, and without their pay or worktime being reduced. Extra staff where scheduled to cover the care aides who would be unable to complete tasks during their interviews.

Semi-structured interviews were designed based on previous literature and covered the following areas: training and entry in LTC, relationships with fellow staff and residents, and experiences of delivering care, including EoL care. Interviews were between 37 and 153 min in duration (average 68 min) and were audio-recorded and transcribed verbatim. Participant recruitment continued until saturation was achieved.

### Data analysis

The data was analysed using a team based reflexive thematic analysis based on Braun and Clarke’s [33–35] analytical steps, as seen in Table 1. Through an iterative process of reading and re-reading the transcripts, codes were constructed using NVivo 10 qualitative analysis software [36] and organised into emergent themes by the first author, who regularly consulted with the other authors in group and individual analyses focused meetings. Themes were refined and then finalised within the research team [33–35].

**Table 1:**
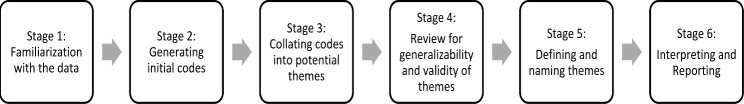
Analyses Process

This was a reflexive team process, where all authors contributed to the reviewing and questioning of the codes, potential themes, and final themes. The analytical process with the first phase involving familiarization with the data. Interviews and field notes were repeatedly read by the first author to become better acquainted and gain an overall understanding of the data. During the second phase, initial codes were generated based on the familiarization of the data from interviews and observations, with field notes being analysed using the same initial themes identified from the interviews. In phase 3, themes were generated by collating codes into potential themes. At this point, some themes were removed, and some were re-collated. Following this, during the fourth phase, themes were reviewed to check whether they related to the coded extracts and to assure internal thematic coherence. In phase 5, themes were defined and named. In phase 6, the findings were written up, and comprehensive themes were created, based on the perspectives and experiences of the participants.

### Trustworthiness

To ensure the data was trustworthy, the criteria set out by Guba and Lincoln [37] The first author gathered and transcribed data verbatim, engaging with the research team in data analysis [38]. Peer-debriefing with research peers specialising in other areas of LTC were regularly undertaken (on average once every three weeks), to ensure assumptions were identified. The research process, including memos and an audit trail, was recorded in a research journal and key issues arising were discussed within the team. Preliminary findings were shared at conferences and seminars to gain further feedback.

## Results

The results from this study are organised into two core themes focused on the care aide’s experiences of delivering EoL care to residents in the LTC home and the supports they feel are necessary to enable them to deliver good quality EoL care. Table 2 shows the themes and subthemes.

**Table 2:** Summary of Results

Theme	Sub Themes
Theme 1: Emotional Toll	Bearing Witness: Distressing Death Experiences (Trigger Warning)
	Relationality in end-of-life care
	Grief: Acclimatization and Isolation
Theme 2: Healing and Support	Accessing and recognition
	Self-care rituals
	A Stigmatized and Ambiguous Role

### Theme 1 emotional toll

#### Bearing witness: Distressing Death Experiences (Trigger Warning)

During the care aide’s education and training, participants reported not being properly prepared to address *distressing* incidents of dying and death, nor being adequately supported after bearing witness to upsetting deaths:

*I don’t think [educators and management] think that we see stuff… There seemed to be very little kind of real-life stuff involved [during education] … when I actually started working as a care aide, it was like this culture shock of…“I did not know it was going to be like this. I didn’t know any of this would happen.”… we just sort of expected everything we would deal with would be sort of easy and peaceful…if someone died, we just find them dead…there wasn’t really any—you’re going to sit there while someone claws at you in terror. [PG034]*

Beyond bearing witness to disturbing deaths, care aides often spoke about the sheer number of deaths that may occur in LTC as being a shock to them. Often participants expressed their most difficult experiences as relating to EoL situations, where the numbers of residents dying was emotionally exhausting.

*Researcher: Do you feel like you’re naturally inclined to work with people who are in palliative care?*

*Participant: In some ways, in some ways. I’ve seen a lot of them in the last couple of years here like the amount of palliative people I met pass away, so it’s a lot. I almost felt like quitting at Christmas time. We lost someone there. And here, say, like the whole floor, we might lose 10 between November and February. And this year in two months, it was like 10 just down my wing….Yes, it was brutal, brutal, brutal, brutal…it was not good…. It’s hard.” [PG035]*

Participants stressed that the times they felt the most likely to leave their role was when many residents died in a short period, and this could place a substantial mental burden on care aides. As seen in the quote above, high numbers of deaths in a short period of time can be “*brutal”* for a care aide.

#### Relationality in end-of-life

Some of the deaths experienced impacted the care aides emotionally more than others because of the close relationships they had developed with the residents. One care aide summed this up as follows:

*An [Indigenous] guy who was …always exit-seeking. … we screwed all his window shut so he can’t open the windows in 24-hour care…the psychologist comes and says, …the money, the funding wasn’t there anymore. That day, [management] took the 24-hour care away. …The next day, he was walking across the street and got hit and killed by a car. …I was freaking mortified.*

*He was only like 48. I used to sit and paint with him. He was my friend. …One manager quit the next day because he couldn’t handle it. He couldn’t handle the way the whole thing -- the way it was all being processed. They just swept it under the mat like, “Don’t talk. We won’t be talking about this.“ … A lot of people quit after that…So, yes, that’s your prime example of a really, really bad [death]. [PG019]*

The mental stress associated with an unexpected, violent death of a resident, who was relationally close to the care aide is highlighted in this excerpt. This participant felt strongly that more could have been done to prevent the death of a resident, whom the participant also described as a “*friend*”. This indicates the close relationality developed between care aide and resident can further exasperate the grieving process. The care aide feeling ‘*mortified’* as well as stifled by an environment of having the experience ‘*swept under the mat’* with a lack of emotional support being received from management and colleagues exacerbating this experience.

*I’d like things to be perfect for them as they’re going to death. I really would. [PG006]*

Although participants shared their care and affection for residents, they also made it clear that the sheer number of residents makes it difficult to constantly develop strong relationships with each one.

*You can’t get attached too much because you’re seeing 20 people in a day or 10. And you have to -- You go as far as you want to go, but you just don’t want to cross the line … you have to have a line drawn where you can’t go too far to drag yourself into another person’s life…. It’s tough. [PG009]*

They describe having the ‘*line drawn*’ as an act of act of conscious self-protection to ensure that they have the capacity to deliver good EoL care to residents, especially when many residents need this support in a short period of time. This was repeated by participants; that to protect their own well-being, they had to build distance and not let a resident death affect them so that they could continue to do their job.

#### Grief: acclimatisation and isolation

Alongside the act of conscious self-protection, participants shared how this notion of ‘*backbone’* and how this was necessary for enduring the amount of deaths and subsequent grief felt by care aides in LTC.

*Being in this field for eight years now, now, you have to just have a backbone, that you have to accept that these people are here for a reason and …they’re not getting younger …So, it’s just accepting for what it is. And that’s how I deal with it. [PG009]*

The “*backbone*” is a tactic for both caring for her own well-being and for facilitating good care to her residents. The juxtaposition of both of these positive goals is that it can disenfranchise the newer care aides that have yet to climatize themselves to the constant reality of the death of their residents. It was not just the actual dying process that care aids described as traumatizing. The procedures involved in ‘after-death’ care was elaborated on by participants as also being a difficult and socially isolating component of their role.

*“When I do my after-death care, I don’t know how I do it but I do what I’m required to do. And then afterwards…when I get home, it’s just like, “Oh, my god, I can’t believe I did that to a dead body.” … it’s hard…I learned [after death care] as I went.... [PG023]*

The key element in this quote is just getting through the situation by holding emotions at bay, these include the grief of the death of a resident and the distress of caring for a dead body, and then reflecting on that experience later with a sense of disbelief. Having to process the experience of delivering after death care when she has left the LTC setting may put her in a position where she is then isolated from others who would have a shared experience (for example, when she is away from fellow care aides and back with family at home). This isolation is a key component of the emotional toll of the care aide role.

The care aides described how they felt they were often put in positions where, when a resident died, they had to care for and attend to the emotional well-being of a grieving family member or close friend of the resident. Care aides reported how they were not trained to deliver grief counselling and not all care aides feel comfortable in this position.

*The care aide is [the] frontline for the family as well. So, you’re dealing with the grieving family…we have no training in how to do that… the family is there when the resident passes…we’re the frontline to support them for their grief… you do your best to console them and everything. And then you kind of just keep going....You put your fake smile on your face. And you pretend like nothing even happened…. There’s no grief counselling. There’s no nothing. There is no memorial thing. Like, there’s no nothing…. No recognition. [PG023]*

This notion of *“just keep going”* is repeated often by care aides. In this revealing account, this participant sheds light on how care aides brace themselves to be able to support the family in their time of grief, yet there is no recognition or space made for the care aides experience of grief. It is when care aides leave the LTC setting that they finally have their own time to grieve.

Alongside there being no known formal mechanisms or pathways to support the care aides’ grief experience in LTC, there was often no established ritual in the LTC institution for recognizing the death of a resident, such as a celebration of life or memorial. In this, both the grief of the care aide and the passing of the resident was not recognized. Participants would sometimes tell their loved ones but often there was a sense of isolation in the grief they were experiencing in their role, as expressed in the following quote:

*Sometimes, I don’t actually tell them the real thing because I don’t think they can handle it.” [PG034]*

The inability to share their experiences and debrief with their loved ones may further disenfranchise the care aides, and the emotional impact of delivering EoL care. The importance of taking care of themselves was varied for care aides, as seen in the following section.

### Theme 2 healing and support

#### Access and recognition

Access to support and resources to facilitate care aides’ mental well-being during the grief process was highlighted as a need by several participants. Feeling alone and unsupported in the delivery of EoL, and during the grieving process, may stem from a disenfranchisement exerted from the care aides who have become desensitized to these experiences:

*There’s people that have troubles with [the death of residents]. I remember my first one. I couldn’t even go in the room… We should have a resource for [grieving] …. Two days ago, I asked this girl why she was off and she said, “I’m new as a care aide and I just couldn’t handle all the deaths on my wing.” And I was like – it’s a shock because I never thought about that. [PG020]*

This care aide recalls how they emotionally struggled with the experience of the first death of a resident. Interestingly, although on one hand she recognizes how this process was difficult for her at the beginning of her career, she is still “*shocked*” that a new care aide would take time off to cope with the deaths on her wing.

As previously described, it was the norm for participants to describe intense stories of bearing witness to violent, unexpected deaths in LTC is an area that care aides highlighted for needing support:

*PG019: [A resident] was going down the hall in his wheelchair, and pop! I found him at the bottom of the stairs dead… …. So, you’re asking me how we dealt with mourning, -- So, the new manager comes upstairs. She says, “How are you?“ And I said -- I just flipped out… She said, “Do you think you can stay and work?“ I’m like -- Yes. And she says like, “Go home for a week on a stress leave.“*

Researcher: did anything happen for you?

*PG019: Nothing. Nothing, zero.*

Researcher: no counselling?

*PG019: Nothing.*

Researcher: nobody checked in on you?

*PG019: Nothing. Zero.*

This care aide felt that nothing was done to support her experience of grief and mental anguish at witnessing the distressing death of a resident. Her “*flipping out*” is an outward expression of processing this extremely upsetting, unexpected situation. Although she describes nothing being done to help her through the grieving process, the manager putting her on stress leave may be seen as supporting her during this time.

Some care aides accessed emotional support from the management team, “*I know that our director of care, her door is open anytime….[PG028]”* but as identified in the previous theme, participants were unaware of any formal resources or mechanisms within the LTC setting to support their well-being around grief and loss.

#### Self-care rituals

As expert carers themselves, care aides shared a myriad of practices developed that shaped their own self-care rituals.

*I read a lot… A friend of mine, she’s a holistic shaman. And she makes these amazing, amazing bath salts… So a bath or two a day and lots of reading. [PG035]*

Other participants shared how a level of self-awareness is needed to recover from EoL care experiences.

*I know I do it my own way like I’ll be really quiet the next hour especially if it’s really that – Yes, and that’s how I know, “Okay this is affecting you,” and there has been days I’ve cried. I go home and I just cry because like all of a sudden it hits you. [PG020]*

The process of waiting till you are home to express emotional anguish may also be a form of self-care, to allow yourself a private, safe place to process what has taken place in the work environment.

*Self-care, it’s tough …It’s just knowing at the end of the day that you’ve done your job the best you can and that’s it…Being at peace because I know I’ve done everything I can to make them comfortable, make them laugh, anything. So, me knowing that makes me better off feeling that I’ve done everything I can. [PG009]*

In the end, participants took solace in knowing that as far as they are capable, they have delivered the best EoL care to their residents as they could.

#### A stigmatized and ambiguous role

Care aides expressed pride in their role of delivering EoL care to residents. As one care aide states, she finds pleasure in ensuring her residents always look their best.

*I like my residents to look great. Even when they’re dying and—You know, like, it’s just one of my most favorite things to do, like, just to make them look comfortable, make them look good. It doesn’t matter if they are dying. [PG025]*

Alongside this, they often shared how the positive aspects of this experience were eclipsed because of society’s view of care aides and how the general public does not know about the true nature of their role. This is evident from the excerpt below.

*[The care aid role has ]been labeled as not good. …. It’s a lower job, it’s a hated job…Yes. When I first like tell my mom, “I want to be a care aide,” I’ve been like frowned upon for quite some time. … “Oh, this is a lower job,“ “This is a dirty job,“ “Nobody want to do that, why would you do that?” But like now, they understand. I’d give them a whole different definition for a care aide -- giving people dignity and giving them the care and whatever you can give them for the very end of their life, right?... So, you’re making them happy before they go. That’s what’s important about being a care aide. [PG036]*

Making a resident “*happy before they go*” is a concise way to redefine the care aide EoL care role.

## Discussion

This study adds to the growing body of international literature taking steps toward understanding the experience of care aides delivering EoL care to residents in LTC in high-income countries. Study findings are consistent with the existing international literature and build on previous research on factors related to gaps in care aide training, in relation to the realities of their role in EoL care [6, 11]. Specifically, this study highlights the trauma affiliated with dealing with residents dying, the lack of care aide preparedness for EoL experiences and the paucity of support that care aides receive regarding the emotional toll EoL care has on their well-being and workplace satisfaction. The findings in this paper outline three areas where changes need to be implemented internationally to support the EoL care experiences of care aides in LTC: at the individual, institutional and societal level.

At the individual level, training must be properly curated to prepare care aides for the role of EoL care delivery. The concept of ‘*just keep going*’ elaborated on in the Emotional Toll theme links with the concept of emotional labour described by Hochschild [39] and the process of ‘*surface acting*’ or ‘*hiding behind your smile*’ as identified by Johnson [40] in her study with care aides working in LTC. In her work on interpretation, practice and emotion in dying in LTC, Funk et al.[41], highlighted the tension between staff members ‘caring about’ a resident, and their lack of time for this work. Despite the recognition that nursing and care professionals bring with them an emotional labour of caring, relatively little international research has been conducted about emotional labour of care aides working in LTC [42] and this study adds to understanding in this area, particularly in relation to the provision of EoL care. Alongside this, participants confirmed that they were asked to perform the role of grief support for deceased residents’ family members. This confirms findings from a recent scoping review of the literature surrounding the concept of EoL care in LTC [2], which found that care aides perform additional tasks outside of what is detailed in their job specification, and that they develop relationships, often familial, with dying residents. There must also be greater care and attention played on the part of the educational settings during their selection and accept process to train care aides to ensure they have previous experience and societal awareness for what care in LTC settings entail, especially regarding EoL experiences.

At the institutional level, the findings from this study indicate a culture change shift is needed in LTC settings internationally to embrace EoL care delivery, and recognize the associated grief as part of the care process, allowing oneself to grieve without guilt or stigmatization. Marcella and Kelley in 2015 [25] interviewed staff members who worked in LTC to gain information on the extent that grief was an issue for them. Similar to this current study, their study found that ‘death is hidden within LTC culture’. There is a lack of training although death is a key part of their role, there are no formal mechanisms put in place for grief, and that support for staff is desperately needed. Despite this study from 2015, and a recent study from 2019 looking at a the emotional experience of staff being in close relationships with residents in LTC [43], culture change surrounding grief support in LTC has a ways to go. A recent review by Gonella et al. [44] looked at what relatives think good EoL care is in LTC. They highlighted how, from the perspective of the relatives, this involves a lot of ‘work’ for staff to deliver this type of EoL care, especially if they have no training in this area. This critical realist evaluation found that, once again, education and inter-professional collaboration are effective at improving EoL care but that staff turnover is a barrier [45]. Another recent review of the literature continues to indicate that managing grief in care work is associated with burnout [46]. Advanced care planning is a way to remove some of the challenges associated with good EoL care to support care aides’ and the family of their residents in preparation for strategic EoL care delivery.

At the societal level, there needs to be a global de-mystifying of the EoL experience, especially in the context within LTC. In this recent review looking at perspectives of people with dementia and there carers on advanced care planning, some of the key points they identified confirm what was found in this current study, including creating a culture of seeing death as part of a journey in LTC, and not something to shy away from embracing and discussing [47]. There is a growing international body of literature around ‘discussing death’ in general society, recognizing that conversations around death and EoL are not common place, disenfranchising the last chapter of life. In recent years though, there are creative mechanisms to push this dialogue forward. Some examples include community interventions such as Death Cafes [48, 49], community interventions that include education and open discussions [50, 51] and incorporating discussions of death in undergraduate university programs [52]. In each of these examples, a core component of the discussion of death may include the experience of death in LTC, with an emphasis on both the resident, and the person delivering the EoL care, the care aide. The difficult experiences of care aides including managing significant excess deaths is documented [53] and the ongoing traumatic impact this may have on care aides remains unknown. National Dementia plans and strategies should include consideration of EoL care in LTC and preparation of the care workforce to effectively support this [54].

In summary, this study has offered significant new insights into the perspectives, experiences, and working conditions of care aides delivering EoL care in LTC. Prior to entry into LTC, the emotional labour associated with the care aide role of delivering EoL needs to be at the forefront of the preparation and training for this vocation. Greater clarity for the role of EoL care needs to be presented to trainees during their preparation for their role in LTC. During LTC employment, the development and enactment of appropriate, inclusive bereavement policies need to be implemented both onsite in LTC facilities, and standardized within international health and care systems to help care aides understand and process emotions and grief surrounding delivering EoL to residents in LTC.

### Limitations

There are two notable limitations of this study. The first being that this is a case study of one specific LTC floor in a more extensive care setting. Although participants in this study spoke about their generalized experiences working as a care aide at an average of 2.5 (range 2–8) different LTC settings, the findings were drawn from their experiences in a range of different facilities and often across extended periods of time. The second limitation concerns the ethnicity of the care aides who chose to participate. In this study, most participants were identified as white. In reality, 33% of care aides in Canada, [55], and 53% of care aides in the US are non-white, [56], and 23% in the UK are immigrants [57]. Considerations of ethnicity must be at the forefront of future LTC research endeavours to ensure that the lived experience of under-served populations in scientific literature is heard and interventions are developed to best support their needs.

## Conclusion

To facilitate the health and well-being of this essential workforce internationally, care aides need to have appropriate training and preparation for the delivery of EoL care and mechanisms put in place to foster their mental health and emotional well-being. Implications for practice highlight the need for greater care and attention played on the part of the educational settings during their selection and accept process to train care aides to ensure they have previous experience and societal awareness for what care in LTC settings entail, especially regarding EoL experiences.

## Data Availability

The datasets generated and/or analysed during the current study are not publicly available due the sensitive nature of the data received but are available from the corresponding author on reasonable request.

## List of abbreviations

LTC: long-term care
EoL: end-of-life
